# Open synovectomy for treatment of tenosynovial giant cell tumors of the knee in children

**DOI:** 10.1093/jscr/rjaf1113

**Published:** 2026-01-20

**Authors:** Hristo Georgiev, Nia Gecheva, Alexander Gerchev, Stefan Tserovski, Daniel-Neno Nenov, Kircho Patrikov

**Affiliations:** Medical University Sofia, University Hospital of Orthopedics “Prof. B. Boychev”, Sofia, Bulgaria; Medical University Sofia, University Hospital of Orthopedics “Prof. B. Boychev”, Sofia, Bulgaria; Medical University Sofia, University Hospital of Orthopedics “Prof. B. Boychev”, Sofia, Bulgaria; Medical University Sofia, University Hospital of Orthopedics “Prof. B. Boychev”, Sofia, Bulgaria; Medical University Sofia, Sofia, Bulgaria; Medical University Sofia, University Hospital of Orthopedics “Prof. B. Boychev”, Sofia, Bulgaria

**Keywords:** tenosynovial giant cell tumor (TGCT), knee, open synovectomy, child

## Abstract

We present seven pediatric cases of open knee synovectomy for tenosynovial giant cell tumor (TGCT)—four diffuse (D-TGCT) and three nodular (N-TGCT). The mean patient age at surgery was 11.3 ± 4.77 years. Five cases were diagnosed and treated within the past 2 years, indicating an unusually high incidence of TGCT in our institution. Subtotal synovectomy was performed through an anterior parapatellar approach, while posterior lesions were accessed between the semitendinosus and medial gastrocnemius muscles. The mean follow-up period was 39 ± 62.3 months (minimum 12 months). No recurrences were detected on follow-up MRI. Six patients regained full knee range of motion, and none exhibited postoperative monoarthritis. These favorable outcomes support open synovectomy as a safe and effective treatment for knee-localized D-TGCT and N-TGCT, especially in cases with posterior or retrocondylar localization.

## Introduction

Tenosynovial giant cell tumors (TGCT) are rare in adults and even less common in children. The neoplasm presents in two clinical forms: diffuse (D-TGCT) and nodular (N-TGCT). The reported incidence in individuals under 18 years of age is 1.30 per 1 000 000 for D-TGCT and 2.86 per 1 000 000 for N-TGCT. The knee joint is most frequently affected in D-TGCT, accounting for ~66% of cases [1]. Until 2013, the condition was referred to as pigmented villonodular synovitis [2, 3].

The literature predominantly describes heterogeneous, isolated pediatric cases of TGCT involving the knee joint [4–7], as well as several more recent reviews of the literature [1, 8–10]. In the pediatric population, surgical excision of the neoplasm—either by open or arthroscopic synovectomy—serves as the therapeutic approach for symptomatic monoarthritis [4, 5, 8, 11–13].

The presented series comprises seven pediatric cases of TGCT treated between 2010 and 2024, five of which were diagnosed and surgically managed within the past 2 years. This unexpected rise in incidence at our institution prompted a detailed analysis of the clinical outcomes following open knee synovectomy.

## Case series

We report four cases of diffuse-type TGCT (D-TGCT) and three cases of nodular-type TGCT (N-TGCT) affecting the knee joint, comprising five female and two male patients, with a mean age at diagnosis and surgical intervention of 11.3 ± 4.77 years (range 4.5–16 years). Surgical management consisted of four subtotal synovectomies and three partial synovectomies—one lateral and two posterior. All patients presented with a painful monoarticular form of knee synovitis. The mean flexion range was limited to 96.42 ± 37.72° (range 30–150°). In three patients, extension was also restricted, with a mean flexion contracture of 8.75 ± 4.78° (range 5–15°). The degree of lesion diffuseness, anatomical localization, and lesion size in the nodular forms are summarized in Table 1, in accordance with the Guide for Diagnosis and Treatment Response Assessment by Spierenburg *et al.* [14].

**Table 1:** Study group patient information^a^

Patient no.	Sex	Age at surgery, y	TGCT type	Preoperative duration of symptoms, mo	Involved joint site size in cm	Knee ROM S-0-0-150°		IntraOP cartilage change outerbridge cl.	Duration of follow-up, mo	Recurrence
						PreOP	PostOP			
										
Case 1	М	4 y, 5 mo	D-TGCT	30	SR+ sup/inf IR + CR + RR lat	0-15-75	0-5-110	IV	24	No
Case 2	F	9 y	D-TGCT	12	SR+ sup/inf IR	0-0-90	0-0-145	I	16	No
Case 3	F	6 y	D-TGCT	36	Sup/inf IR + CR	0-10-110	0-0-145	I	12	No
Case 4	F	13 y	N-TGCT	12	RR med 3/1/2 cm	0-0-120	0-0-150	I	12	No
Case 5	F	16 y	N-TGCT	3	RR med 3/2/2 cm	0-0-150	0-0-150	0	14	No
Case 6	М	15 y	N-TGCT	1	SR 4/2/2 cm	0-0-100	0-0-150	0	15	No
Case 7	F	15 y, 6 mo	D-TGCT	7	SR+ sup/inf IR	0-5-30	0-0-145	0	180	No

^a^CR, central synovial recess; ROM, range of motion; RR, retrocondylar or posterior femoral recesses (medial and lateral); SR, suprapatellar recess; Sup/inf IR, superior and inferior infrapatellar recesses.

### Surgical tehnique

**Figure 1:**
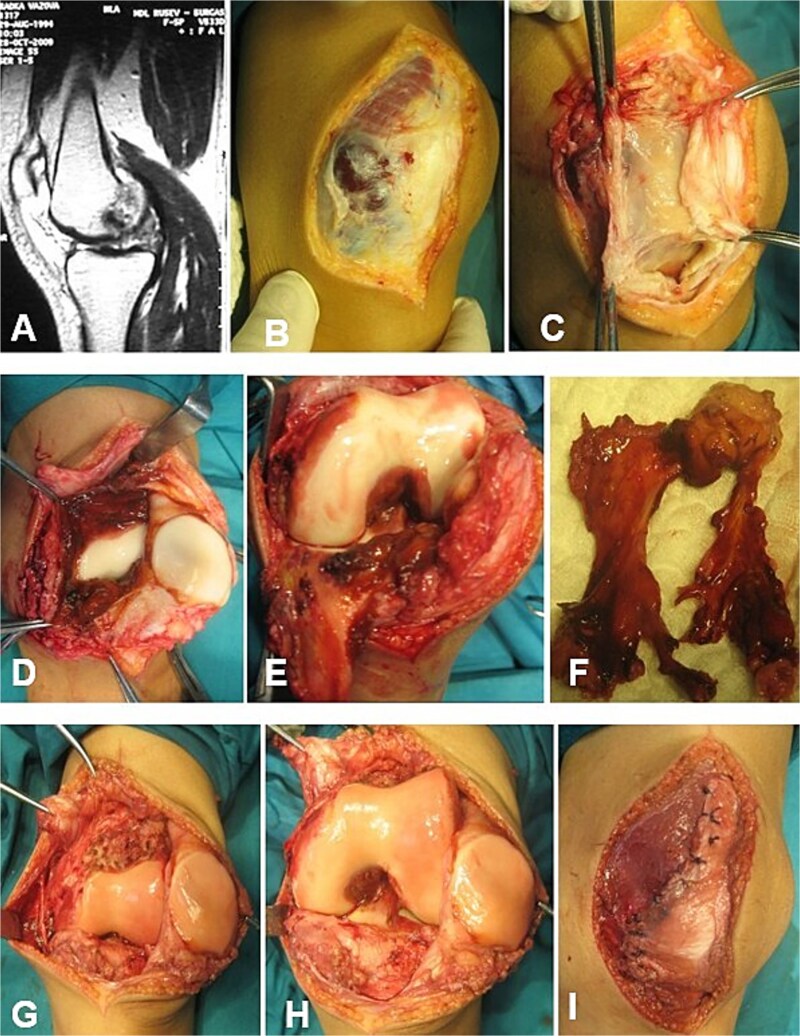
Open synovectomy for D-TGCT of the left knee in a 15-year-and-6-month-old female patient. Case 7. (A–C) Medial parapatellar approach. The neoplasm becomes clearly visible following incision of the fibrous capsule. (D–F) Extensive involvement of the SR, sup/inf IR, and CR. (G and H) Articular surfaces following sharp and electrocautery-assisted removal of the pathological synovium. All accessible and visibly affected areas, including those surrounding the cruciate ligaments, were completely excised. The cruciate ligaments and menisci remained intact. (I) Restoration of the fibrous capsule. CR, cruciate recess; SR, suprapatellar recess, sup/inf IR, superior/inferior infrapatellar recesses.

In cases of D-TGCT, a medial parapatellar approach was utilized, providing full direct visualization of the synovium in the suprapatellar pouch, medial compartment, and—following patellar dislocation—the lateral compartment (Fig. 1B–D). The pathologically altered synovium was circumferentially excised with blunt dissection, starting from the medial tibia and staying within healthy margins. With the knee in flexion, residual pigmented nodules around the cruciate ligaments were visualized (Fig. 1E) and removed either sharply or by meticulous electrocauterization. The fibrous capsule and ligamentous structures were preserved (Fig. 1G–H). This approach allows radical synovectomy, except for small posterolateral and posteromedial areas of the joint (Fig. 1F), where complete resection is limited by anatomical constraints.

For posteriorly localized N-TGCT, a longitudinal posterior approach was employed along the line of the hamstring muscles. In cases of medial nodular localization, the m. gastrocnemius medialis was retracted toward the popliteal fossa to protect both the main neurovascular bundle and its own innervation and vascular supply. After incision of the fibrous capsule, the tumor was carefully excised within healthy synovium (Figs 5C–E and 6C). For lateral localization, partial detachment of the m. gastrocnemius lateralis was performed, with careful protection of the n. peroneus.

**Figure 2:**
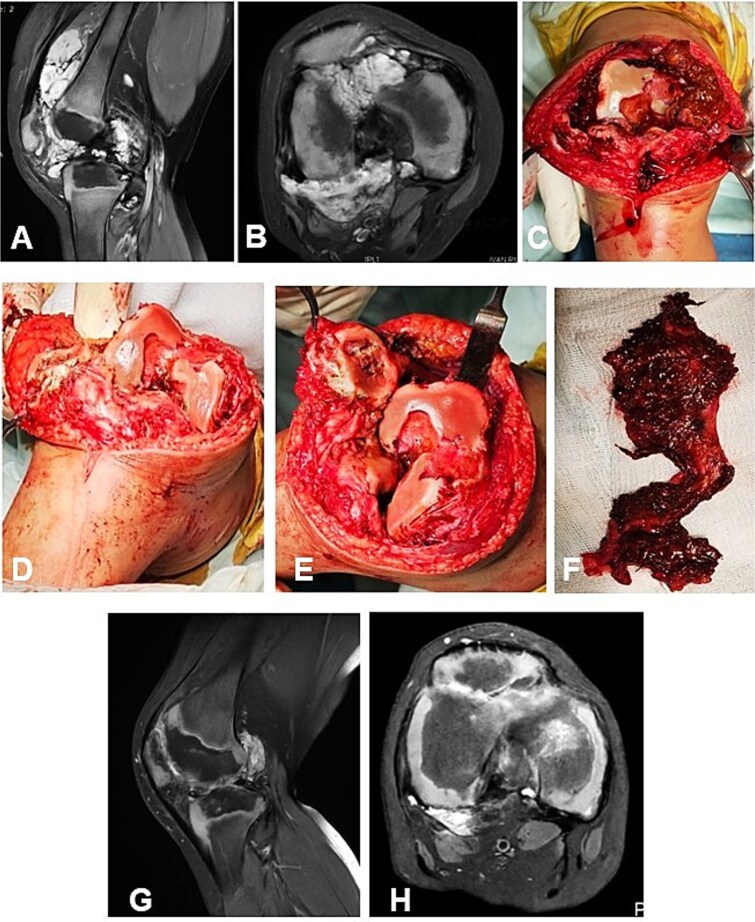
Case 1. (A and B) Preoperative T1 sagittal and axial MRI sequences with PD FS. (C and F) Macroscopic appearance of the pathological synovium before and after its excision. (D and E) The knee joint following synovectomy, demonstrating intact menisci and cruciate ligaments. Chondral lesions correspond to Outerbridge grade IV. (G and H) Postoperative 24 m. Sag and Ax MRI PD FS. No evidence of recurrence of the resected neoplasm was detected. Residual TGCT involvement persists within the lateral posterior femoral recesses. PD FS, proton density weighting with fat suppression; TSE, turbo spin echo.

Joint surface areas infiltrated by synovial pannus were gently curetted (Fig. 2C–E). Following release of the Esmarch tourniquet, hemostasis was achieved, and closure was performed in layers, including repair of the fibrous capsule, subcutaneous tissue, and skin (Figs 1I and 5E). Histopathology of the excised synovium confirmed the diagnosis. Postoperatively, patients were immobilized with a hinged knee brace and underwent physiotherapy with progressive flexion gain of ~15° per week.

### Representative cases

#### Case 1

A 4-year-and-5-month-old child presented with a history of chronic synovitis of the right knee joint persisting for more than 2 years. Progressive limitation of motion was observed—a 15° flexion contracture, and maximum flexion limited to 75°. Magnetic resonance imaging (MRI) was concordant with the intraoperative findings, demonstrating diffuse involvement of both anterior and posterior recesses of the knee joint (Fig. 2A–C). A subtotal anterolateral synovectomy was performed. Intraoperatively, chondral defects corresponding to Outerbridge grade IV were identified (Fig. 2C–E). At 2-year follow-up, a substantial improvement in the range of motion was achieved (Table 1). Follow-up MRI demonstrated no evidence of local recurrence in the previously resected area of the neoplasm (Fig. 2G–H). A secondary synovectomy is planned to address residual synovial involvement within the posterior lateral femoral recess.

#### Case 2

**Figure 3:**
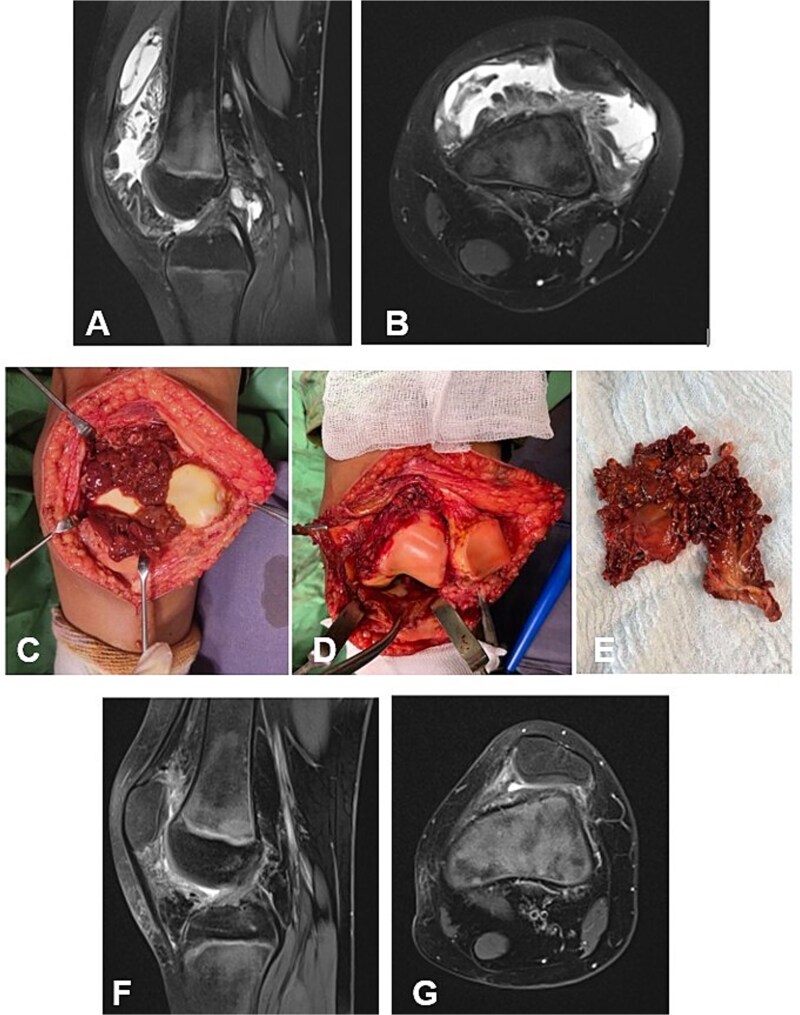
Case 2. (A and B) Preoperative Sag and Ax MRI weighted TSE PD FS. (C and E) Macroscopic appearance of the pathological synovium before and after its excision. (D) The knee joint following synovectomy. Chondral lesions correspond to Outerbridge grade I. (F and G) Postoperative 16 m. Sag and Ax MRI TSE PD FS—no evidence of recurrence of the resected neoplasm. Persistent bone marrow edema, though without clinical symptoms. PD FS, proton density weighting with fat suppression; TSE, turbo spin echo.

A 9-year-old female presented with a one-year history of knee pain, swelling, and progressive limitation of flexion, unresponsive to nonsteroidal anti-inflammatory drugs and physical therapy. MRI demonstrated a large anterior synovial mass without evidence of bone erosion (Fig. 3A and B). The D-TGCT involved the suprapatellar recess as well as the superior and inferior infrapatellar recesses (Fig. 3C). The pathologically altered synovium was excised en bloc through an open synovectomy (Fig. 3D and E). The hyaline cartilage changes were classified as Outerbridge grade I. At 18 months postoperatively, the patient was asymptomatic, exhibiting full range of motion in the knee. Follow-up MRI revealed no evidence of recurrence of the resected neoplasm (Fig. 3F and G).

#### Case 3

**Figure 4:**
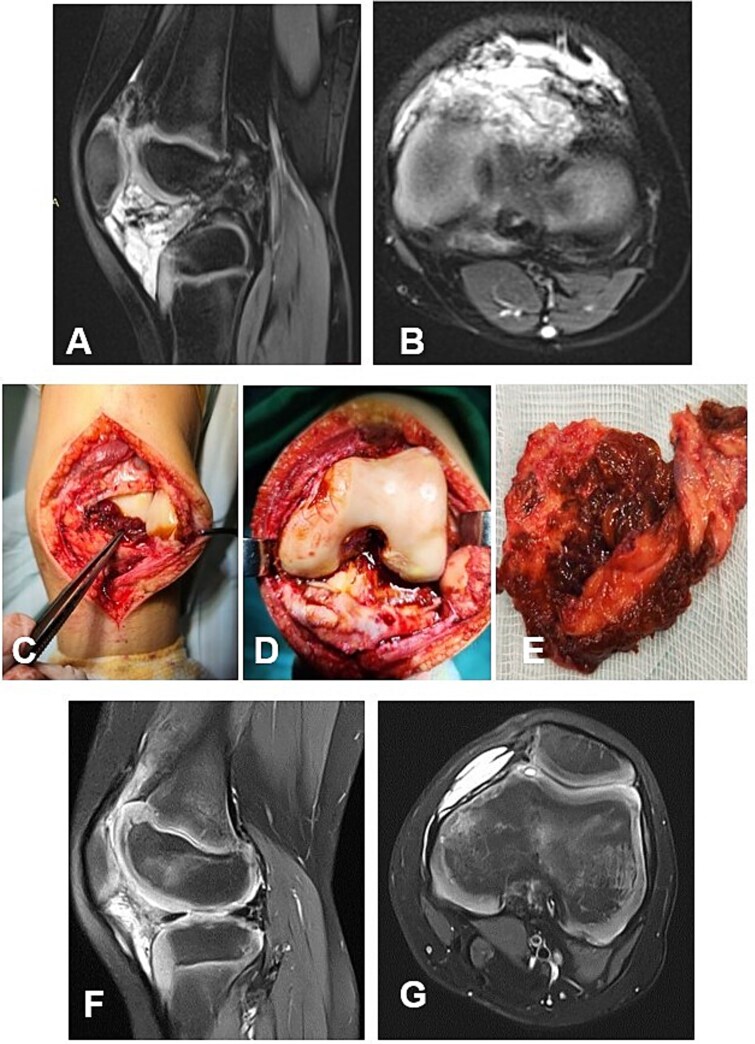
Case 3. (A and B) Preoperative sag and ax MRI TSE PD FS. (C and E) Macroscopic appearance of the pathological synovium before and after its excision. (D) The knee joint following synovectomy. Chondral lesions correspond to Outerbridge grade I. (F and G) Postoperative 12 m. Sag and Ax MRI TSE PD FS—no evidence of recurrence of the resected neoplasm. PD FS, proton density weighting with fat suppression; TSE, turbo spin echo.

A 6-year-old girl with a 3-year history of knee pain and swelling, previously treated for Synovitis transitoria. The chronic course of the condition corresponded to progressive limitation of flexion and the development of a flexion contracture (Table 1). The diagnosis was established by MRI (Fig. 4A and B). The D-TGCT involved the central, superior, and inferior infrapatellar synovial recesses (Fig. 4C). Through open synovectomy, the visibly altered synovium was excised en bloc (Fig. 3D and E). The hyaline cartilage changes were classified as grade I according to Outerbridge. One year after surgery, the child remained asymptomatic with a full range of motion in the knee. MRI showed no evidence of recurrence of the excised neoplasm (Fig. 3F and G).

#### Case 4

**Figure 5:**
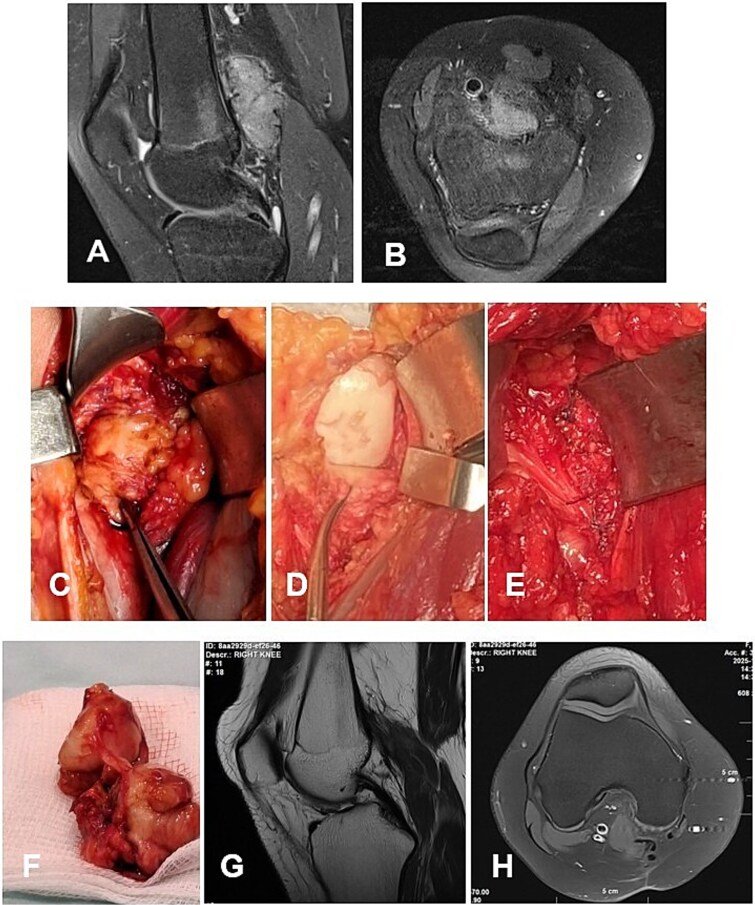
Case 4. (A and B) Preoperative Sag and Ax MRI TSE PD FS—a lobulated intra-articular neoplasm with a posterior mediocentral localization, displacing the neurovascular bundle laterally. (C and F) Macroscopic appearance of the N-TGCT measuring 3 × 1 × 2 cm, *in situ* and after excision. (C) Posterior synovectomy performed with lateral retraction of the *m. gastrocnemius medialis.* (D) The joint after synovectomy, showing grade I chondral changes according to the Outerbridge classification. (E) Reconstructed fibrous capsule. (E and F) Postoperative 12-month sagittal and axial MRI (TSE PD FS) demonstrates no evidence of recurrence of the resected neoplasm. (G and H) PD FS, proton density weighting with fat suppression; TSE, turbo spin echo.

A 13-year-old female patient presented with a nodular tenosynovial giant cell tumor (N-TGCT) involving the medial posterior femoral recess. The clinical course was characterized by intermittent knee pain and swelling persisting for ~12 months. MRI demonstrated an intra-articular, well-defined lobulated lesion with posterior mediocentral localization, causing lateral displacement of the neurovascular bundle (Fig. 5A and B). A complete excision of the lesion was achieved through a posterior synovectomy, performed sharply and within macroscopically healthy synovial margins (Fig. 5C and D). At 12-month postoperative follow-up, the patient was asymptomatic, with full range of motion and no MRI evidence of local recurrence. The ligamentous apparatus remained intact.

#### Case 5

**Figure 6:**
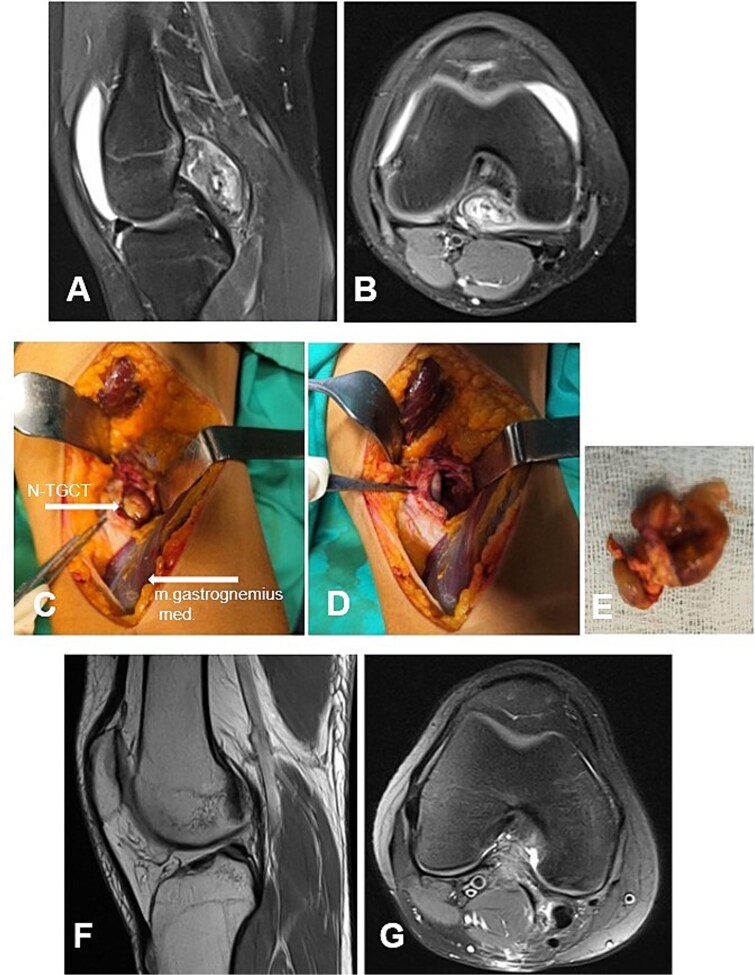
Case 5. (A and B) Preoperative sag and ax MRI TSE PD FS—lobulated edematosus intra-articular neoplasm with a posterior mediocentral localization. (C and D) Posterior synovectomy between m. semitendinosus and m. gastrognemius medialis. (Е) Macroscopic appearance of the N-TGCT measuring 3 × 2 × 2 cm after excision. (F and G) Postoperative 12 m. Sag and Ax MRI TSE PD FS—no evidence of recurrence of the resected neoplasm. PD FS, proton density weighting with fat suppression; TSE, turbo spin echo.

А 16-year-old female athlete presented with knee effusion and pain without preceding trauma, persisting for 3 months. Diagnostic MRI revealed an intra-articular, retrocondylar mass with intact cruciate ligaments and menisci (Fig. 6A and B). The lesion was identified as a nodular tenosynovial giant cell tumor and was excised through a posterior partial synovectomy using a medial approach with lateral retraction of the m. gastrocnemius medialis. The tumor was removed sharply and en bloc within macroscopically healthy synovial margins (Fig. 6C–E). At the 12-month postoperative follow-up, the patient was asymptomatic, with no MRI evidence of local recurrence (Fig. 6F and G). Successful return to full athletic activity.

## Discussion

The cases presented in our series corroborate previously published data regarding the incidence and age distribution of TGCT of the knee in the pediatric and adolescent population [1, 8, 11]. An apparent increase in case frequency after 2022 was observed, the etiology of which remains uncertain. A diagnostic delay was noted in the majority of cases, with a mean latency period of 16 ± 12.5 months (range, 1–36 months) between symptom onset and definitive diagnosis by MRI. The suspended diagnosis was due to non-specific clinical manifestations, equivocal physical findings, and inconclusive results from conventional imaging modalities [1, 8, 10]. Ultrasonography typically showed variable intra-articular effusion with irregular hypoechoic synovial thickening, while radiographs and laboratory tests were normal. MRI was considered the diagnostic modality of choice. In diffuse-type TGCT, MRI typically demonstrates diffuse, irregular synovial thickening involving the joint recesses, often with nodular and villous projections and associated joint effusion. The signal characteristics are typically low on T1-weighted and high on Short-TI Inversion Recovery (STIR) sequences. Areas of low signal intensity on T2-weighted images correspond to hemosiderin deposition within the synovium and nodules [1]. A direct correlation was observed between the duration of the process and the development of subchondral erosive changes (Table 1). Based on these findings, we advocate MRI evaluation in all pediatric patients presenting with monoarticular knee effusion persisting for more than 30 days.

The open subtotal synovectomy approach proposed by our team for the treatment of TGCT of the knee in pediatric patients provides wide surgical exposure, direct visual control, and enables maximal resection of D-TGCT. No local recurrence was observed on serial MRI at a mean follow-up of 39 ± 62.3 months (minimum, 12 months). Furthermore, no episodes of recurrent postoperative monoarthritis were observed. Full range of motion of the knee joint was restored in six patients, and the level of satisfaction among the children and their parents regarding postoperative functional and cosmetic outcomes was very high.

Subtotal synovectomy does not allow for complete removal of the pathological synovium when the entire synovial membrane is affected. In such cases, a secondary posterior approach may be required, either during the same procedure or at a later stage. In our series, no secondary posterior synovectomies were performed. In Case 1, despite marked clinical improvement, a planned posterior synovectomy is scheduled due to the extensive posterior localization of the lesion. In the remaining patients, small, asymptomatic residual foci of TGCT detected on postoperative MRI were left in situ and are being closely monitored through active surveillance.

An alternative to the open technique is arthroscopic synovectomy, which offers several recognized advantages, including minimized surgical trauma, cosmetic results, and a shorter postoperative rehabilitation period [10, 12, 15]. These benefits, however, are considered applicable primarily to nodular-type TGCT (N-TGCT) with anterior localization. In contrast, for the diffuse-type TGCT with retrotrochlear or posterior recess involvement, the open synovectomy approach provides superior visualization and access, thereby allowing more complete resection of the neoplastic synovium and resulting in a lower likelihood of local recurrence [1, 16]. The surgical exposures utilized in our series were carefully selected to preserve the vascularization and innervation of both the extensor mechanism and the medial head of the gastrocnemius muscle. Despite this anatomical preservation, structured postoperative physiotherapy, emphasizing a gradual increase in range of motion, remains essential for optimal functional recovery of the knee joint.
